# Improvised peritoneal dialysis in an 18-month-old child with severe acute malnutrition (kwashiorkor) and acute kidney injury: a case report

**DOI:** 10.1186/1752-1947-7-168

**Published:** 2013-06-28

**Authors:** Francis Fredrick, Gudila Valentine

**Affiliations:** 1 Department of Paediatrics and Child Health, School of Medicine, Muhimbili University of Health and Allied Sciences, PO Box 65001, Dar es Salaam, Tanzania; 2 Renal Unit, Muhimbili National Hospital, PO Box 65000, Dar es Salaam, Tanzania

## Abstract

**Introduction:**

Severe acute malnutrition is common in developing countries. Children with severe acute malnutrition are prone to complications, including electrolyte imbalance and infections. Our patient was an 18-month-old boy who had severe acute malnutrition (kwashiorkor) and developed acute kidney injury, which was managed with peritoneal dialysis using improvised equipment. This case report illustrates the importance of improvisation in resource-limited settings in providing lifesaving treatment. To the best of our knowledge, this is the first case report on peritoneal dialysis in a child with severe acute malnutrition (kwashiorkor).

**Case presentation:**

We report a case of an 18-month-old Bantu-African Tanzanian boy who had severe malnutrition and developed anuric acute kidney injury. He had severe renal dysfunction and was managed with peritoneal dialysis using an improvised catheter and bedside constituted fluids (from intravenous fluids) and was diuretic after 7 days of peritoneal dialysis, with complete recovery of renal functions after 2 weeks.

**Conclusion:**

Children with severe acute malnutrition who develop acute kidney injury should be offered peritoneal dialysis, which may be provided using improvised equipment in resource-limited settings, as illustrated in this case report.

## Introduction

Severe acute malnutrition is common in children in developing sub-Saharan Africa, and children with severe malnutrition are prone to several complications. We present a case of an 18-month-old boy who had severe acute malnutrition (kwashiorkor) and developed anuric acute kidney injury, which was successfully managed with peritoneal dialysis (PD) using an improvised catheter and modified PD fluid with intravenous fluids. This case highlights the role of PD in the treatment of acute kidney injury in small children, as well as the role of improvising and using available resources in performing PD in limited-resource settings.

## Case presentation

An 18-month-old Bantu-African Tanzanian boy was presented to our hospital with severe acute malnutrition (kwashiorkor). The patient presented with weight loss of 3 months’ duration and generalized body swelling for the previous 3 weeks. He also had had watery diarrhea and fever for 1 day prior to admission. At admission, the child was afebrile with a pulse rate of 100 beats/min and a respiratory rate of 28 breaths/min. He had severe palmar pallor, puffy face, and pedal edema with peeling of skin in the lower limbs. The child’s weight was 8.2kg, length was 78cm, and mid-upper-arm circumference was 11cm. His weight for length was between a –2 standard deviation (SD) and –3SD *z*-score according to World Health Organization growth standard charts. He had hepatomegaly of 3cm below the right lower costal margin. The diagnoses of kwashiorkor, acute watery diarrhea, and severe anemia (not in anemic heart failure) were made. His serum albumin was 13g/L, serum creatinine was 30.5μmol/L, serum sodium 129mmol/L, and serum potassium 3.6mmol/L. The complete blood count revealed Hb of 8.5g/dl, mean corpuscular volume 52.5fl, mean corpuscular Hb 16.2pg, and white blood cell count 9.07 × 10^3^/μL. Urinalysis was normal, blood slide had no malaria parasite, and serology screening for human immunodeficiency virus, hepatitis B virus, and hepatitis C virus was normal. The child was treated with special diet Formula 75, low osmolarity oral rehydration solution, pediatric zinc, folic acid, and vitamin A. The child was put on the intravenous antibiotics ampicillin, cloxacillin, and gentamicin for 7 days.

The patient’s diarrhea stopped on his fourth day in the ward, edema of his lower limbs subsided, and he had no fever. On the seventh day postadmission, the boy’s Formula 75 diet was changed to Formula 100. On the ninth day after his admission, he had reduced urine output (24-hour urine output 20ml), no fever, no hematuria, and no skin infection, but he had a reappearance of puffy face. Furosemide injection was given to challenge the kidneys, which brought no improvement. The child was anuric on the tenth day postadmission. A diagnosis of acute anuric kidney injury was made. His serum creatinine was 85.7μmol/L, serum potassium was 4.1mmol/L, and serum sodium was 132mmol/L. Daily input was restricted to 500ml, the child was monitored with serial serum creatinine and electrolytes, and he was anuric for a total of 5 days, after which PD was started. Before PD, his serum creatinine was 256μmol/L, serum potassium was 4.7mmol/L, and sodium was 127mmol/L.

**Figure 1:**
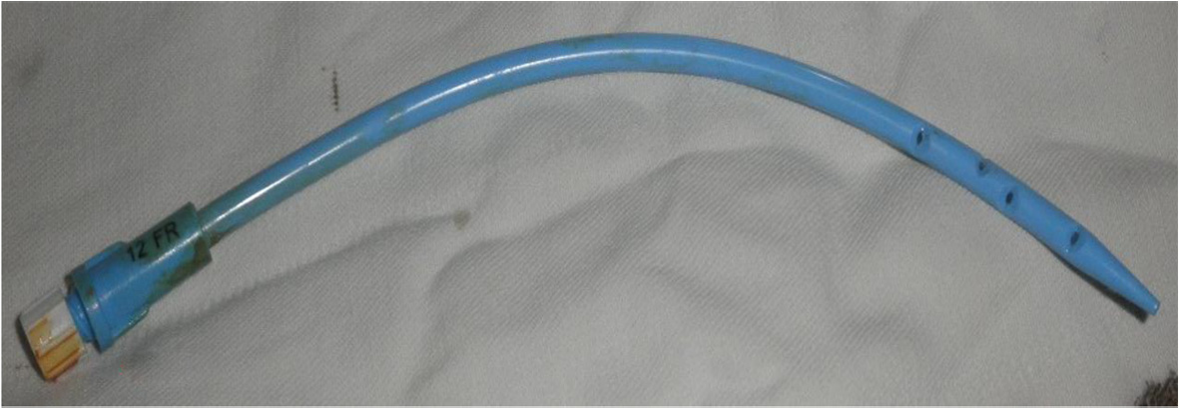
Suprapubic aspiration catheter modified into a peritoneal dialysis catheter by cutting holes in the distal part of the catheter.

**Figure 2:**
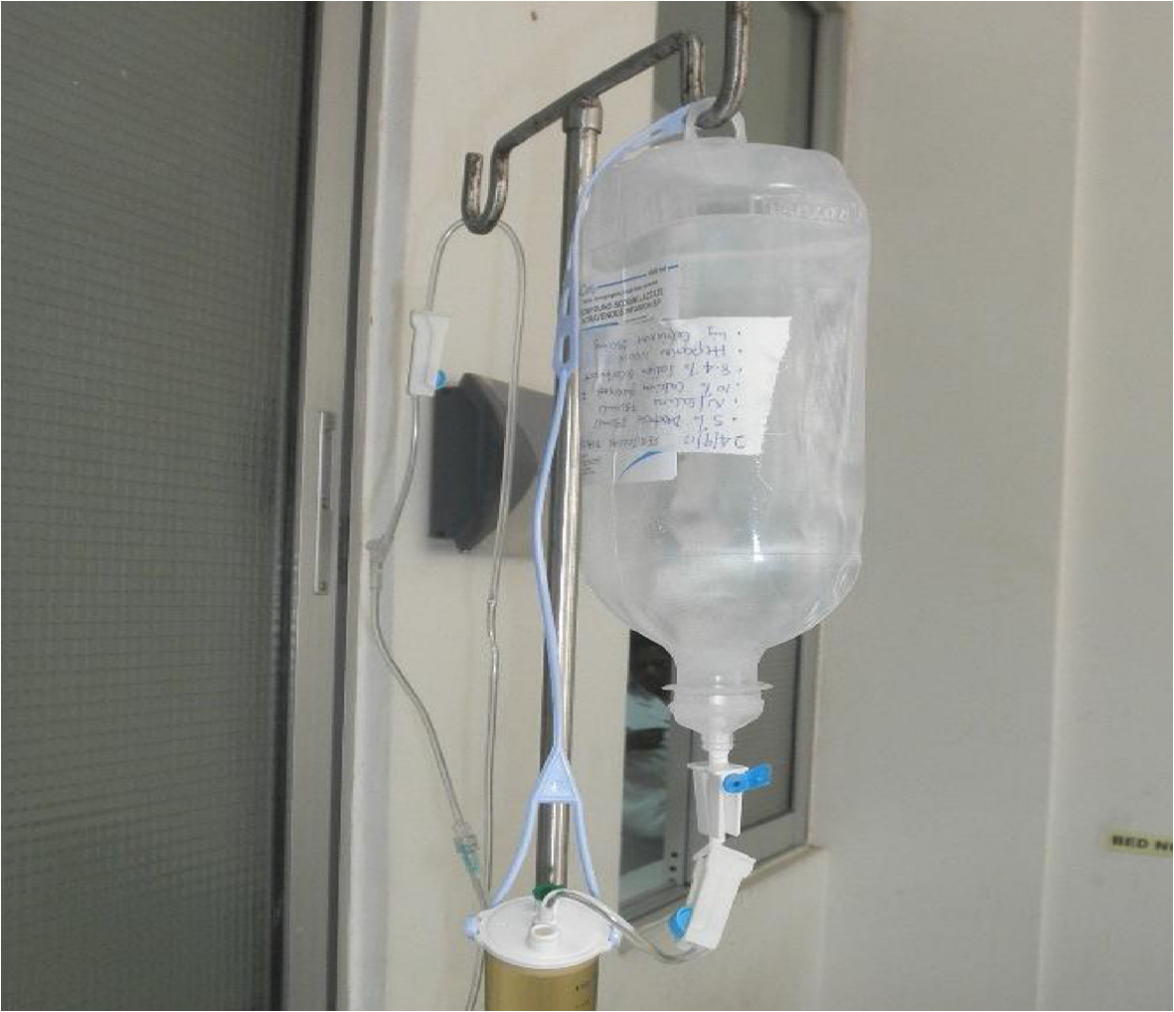
Peritoneal dialysis fluid made at bedside using 250ml of 5% dextrose solution, 750ml of 0.9% normal saline, 40ml of 8.4% sodium bicarbonate, 7.5ml of 10% calcium gluconate, 1000U of heparin, and 250mg of ceftriaxone.

PD was carried out using a suprapubic aspiration catheter as a PD catheter; this was modified by cutting small side holes (Figure 1). The modified catheter was introduced into the peritoneal cavity after making a small incision and dissection of tissues to the rectus sheath under aseptic conditions, and this procedure was performed under local anesthesia using 2% lignocaine. PD fluid was made at bedside using 250ml of 5% dextrose solution, 750ml of 0.9% normal saline, 40ml of 8.4% sodium bicarbonate, 7.5ml of 10% calcium gluconate, 1000 units of heparin and 250mg of ceftriaxone (Figure 2). Dwell time was 30 minutes for the first exchange and 2 hours for subsequent 2 exchanges and 6 hourly thereafter. Dwell volumes were 250ml for the first two dwells, and was reduced to 200ml for subsequent dwells because of leakage. The child was also given ceftriaxone injection 500mg once daily at initiation of PD. Serum creatinine peaked at 384.6μmol/L on day 3 of PD, and serum electrolytes were within normal range. The child was kept on PD for 7 days, and he started to pass urine (20ml) on the 4^th^ day after starting PD, on the 5^th^ day urine output increased to 80ml and on the 6^th^ day the child passed 340ml of urine. The PD catheter was removed on the 7^th^ day after starting PD.

The child’s condition improved after PD, and his serum creatinine level decreased gradually. The child was continued on the Formula 100 diet and was given ferrous sulfate tablets for iron deficiency anemia. An abdominal ultrasound was performed after stopping PD, which revealed normal-sized kidneys with normal cortical medullary differentiation. The patient was discharged 2 weeks after PD was stopped, at which time his serum creatinine was 41. 4μmol/L. The child was seen 2 weeks later in the follow-up clinic and was doing fine, and his serum creatinine was 32.8μmol/L.

## Discussion

Severe malnutrition is common in children, especially in sub-Sahara Africa, and these children are susceptible to various complications, including hypoglycemia, hypothermia, electrolyte imbalance, and infections [1-3]. Children with severe acute malnutrition are treated in phases, with the first phase being stabilization with an emphasis on treating infections and replenishing the deficient micronutrients. In this phase, the patients are mainly given a Formula 75 diet, which is low in energy and protein contents [1]. After stabilization, patients are fed a Formula 100 diet, which is rich in energy and protein for catch-up growth [1].

In the present case report, we describe a peculiar case of severe acute malnutrition complicated by severe acute kidney injury. The cause of acute kidney injury could not be clearly identified but was attributed to various factors, including infection and nephrotoxic drugs (especially gentamicin) which the patient had received. Acute kidney injuries in children are usually multifactorial [4].

Our patient developed anuric acute kidney injury, and the patient was successfully managed with acute PD using an improvised catheter (Figure 1) and PD fluids which were prepared at bedside using intravenous fluids (Figure 2). PD is the modality of choice in managing acute renal failure in children [5], with the advantages of being easy to perform without requirement of sophisticated equipment as compared to hemodialysis [6]. The outcome of children with acute kidney injury treated with PD has been reported to be remarkably good [7,8].

Access to PD is universal in developed countries but not as prevalent in developing countries [9]. Initiatives to establish PD in resource-limited settings have proved to be successful, as reported by Callegari *et al.* on PD for treatment of acute kidney injury in Kilimanjaro, Tanzania [10]. In our hospital, we had no acute PD catheter or fluids, which necessitated this improvised technique in managing acute kidney injury. Our patient had kwashiorkor and developed acute kidney injury, and he was treated with improvised PD as a modality of renal replacement therapy, which led to a good outcome. A similar approach was reported by Obiagwu *et al.* in a child with acute kidney injury in Kano, Nigeria [11]. This shows that a patient with kwashiorkor who develops acute kidney injury may benefit from renal replacement therapy in the form of PD.

## Conclusion

Children with severe malnutrition who develop acute kidney injury should be offered PD as a modality of renal replacement therapy. In resource-limited settings, lifesaving PD may be provided by improvising locally available equipment and intravenous fluids, as illustrated in this case report.

### Consent

Written informed consent was obtained from the patient’s legal guardian for publication of this manuscript and accompanying images. A copy of the written consent is available for review by the Editor-in-Chief of this journal.

## Abbreviations

HBV: Hepatitis B virus; HCV: Hepatitis C virus; HIV: Human immunodeficiency virus; MCH: Mean corpuscular hemoglobin; MCV: Mean corpuscular volume; SD: Standard deviation; WHO: World Health Organization.

## Competing interests

The authors declare that they have no competing interests.

## Authors’ contributions

FF analyzed and interpreted patient data and wrote the manuscript. GV gathered patient data and participated in its analysis. Both authors read and approved the final manuscript.
